# Metabolomics and partial least square discriminant analysis to predict history of myocardial infarction of self-claimed healthy subjects: validity and feasibility for clinical practice

**DOI:** 10.1186/s13336-015-0018-4

**Published:** 2015-03-13

**Authors:** Nornazliya Mohamad, Rose Iszati Ismet, MohdSalleh Rofiee, Zakaria Bannur, Thomas Hennessy, Manikandan Selvaraj, Aminuddin Ahmad, FadzilahMohd Nor, ThuhairahHasrah Abdul Rahman, Kamarudzaman Md.Isa, AdzroolIdzwan Ismail, Lay Kek Teh, Mohd Zaki Salleh

**Affiliations:** Integrative Pharmacogenomics Institute (iPROMISE), Universiti Teknologi MARA (UiTM), Bandar Puncak Alam, Puncak Alam Malaysia, Selangor 42300 Malaysia; Life Sciences & Diagnostics Group, Translational Research Institute, Brisbane, Australia; Faculty of Medicine, Universiti Teknologi MARA (UiTM), Sungai Buloh, Selangor Malaysia; Faculty of Pharmacy, Universiti Teknologi MARA (UiTM), Bandar Puncak Alam, Selangor 42300 Malaysia

**Keywords:** Orang Asli, Metabolomics, Myocardial infarction, Predictive model, Phenotype

## Abstract

**Background:**

The dynamics of metabolomics in establishing a prediction model using partial least square discriminant analysis have enabled better disease diagnosis; with emphasis on early detection of diseases. We attempted to translate the metabolomics model to predict the health status of the Orang Asli community whom we have little information. The metabolite expressions of the healthy vs. diseased patients (cardiovascular) were compared. A metabotype model was developed and validated using partial least square discriminant analysis (PLSDA). Cardiovascular risks of the Orang Asli were predicted and confirmed by biochemistry profiles conducted concurrently.

**Results:**

Fourteen (14) metabolites were determined as potential biomarkers for cardiovascular risks with receiver operating characteristic of more than 0.7. They include 15S-HETE (AUC = 0.997) and phosphorylcholine (AUC = 0.995). Seven Orang Asli were clustered with the patients’ group and may have ongoing cardiovascular risks and problems. This is supported by biochemistry tests results that showed abnormalities in cholesterol, triglyceride, HDL and LDL levels.

**Conclusions:**

The disease prediction model based on metabolites is a useful diagnostic alternative as compared to the current single biomarker assays. The former is believed to be more cost effective since a single sample run is able to provide a more comprehensive disease profile, whilst the latter require different types of sampling tubes and blood volumes.

**Electronic supplementary material:**

The online version of this article (doi:10.1186/s13336-015-0018-4) contains supplementary material, which is available to authorized users.

## Introduction

Cardiovascular disease (CVD) is known as the major cause of death worldwide [1]. According to the World Health Organization report in 2013, 17.3 million deaths were estimated; and the majority were due to ischemic heart disease and stroke. The National Health and Nutrition Examination Survey of United States of America reported that from 2007 to 2010, the incidence of CVD in adults was found to increase with age [2]. According to the Department of Statistics Malaysia, this non-communicable disease has become one of the major causes of death with increasing percentage from the year 2005 to 2008. The National Health and Nutrition Examination Survey of the United States of America also predicted that the number of people who died from CVD would be 23.3 million by 2030, worldwide.

Lipid measurements including the concentrations of high-density lipoprotein cholesterol (HDL) and low-density lipoprotein cholesterol (LDL) were routinely used as indicators to assess cardiovascular risks [3]. Smoking, genetics, overweight and high carbohydrate uptake affect both the HDL and LDL levels [4]. Mora*et al*. [5] found that HDL level was consistently and inversely associated with the incidence of coronary and cardiovascular events across a range of LDL, where high level of LDL caused higher risk of CVD. The lipid parameters however provided a narrow view of the lipid metabolism as reported in the previous study [3-5]. Alternatively, real time monitoring of the validated biomarkers comprised of differentially expressed metabolites of a patient is desirable in order to allow more accurate and early detection.

Metabolomics is one of the core disciplines of systems biology. It has been integrated into multidisciplinary approaches to profile changes in small molecules associated with human diseases. Metabolites are the end-products of gene expression, which is closely related to protein and enzymatic reactions. Therefore, metabolites offer a direct molecular signature of status of cells that reflect changes in the phenotype and molecular physiology and are useful for evaluation of the efficacy of medical treatments [6,7]. Recent innovations in instrumentation, bioinformatics tools and softwares have enable comprehensive analysis of cellular metabolites and therefore enhance the potential of metabolomics to be adopted for clinical diagnostics and industries [8,9]. Advanced techniques, such as NMR, GCMS/QTOF and UPLCMS/QTOF have allowed researchers to determine biomarkers for detection and monitoring of the progress of diseases using urine and serum samples [10-12]. The dynamics of metabolomics have enabled a comprehensive real time understanding on the phenotypic changes relating to disease states, accounting the influence of variations in genes and proteins, epigenetic regulation and post-translational modifications. For examples, the changes of 5-OH-tryptophan, 2-OH-butyric acid and 3-OH-butyric acid, which are involved in hypoxia, are useful for the early detection of acute coronary syndrome (ACS) because often time ACS is detected when the disease is in an advanced and irreversible stage [13].

The Orang Asli is the indigenous people of Peninsular Malaysia. They are believed to be among the first people to have arrived in Peninsular Malaysia by different waves of migration back to about 50,000y years before present [14]. They constitute 1.5% of the Malaysian population and is relatively small in numbers compared to the other Malaysian ethnics [15]. One of the major concerns of the Orang Asli population is their numbers are declining. This leads to the question of what had caused the declining in numbers of these populations in Peninsular Malaysia. Over the past three decades, the Orang Asli had undergone relocation programs by the Government of Malaysia. These relocation programs provided the Orang Asli with better amenities and promises for modern infrastructure. However, with these relocation programs, the Orang Asli has scant access to foraging, fishing, as well as agriculture resulting in life styles that are more sedentary. This sedentary way of living may have influenced their health status. The changes of environmental and lifestyles may bring evolutionary adaptive changes from total dependence on forest resources to reliance on the market economy [16]. Reports on the health status of the Orang Asli are also limited due to the difficulty in obtaining voluntary participation of the Orang Asli in research. The health quality of the Orang Asli was reported to be poorer as compared to the urban people because of the nutritional deficiency and poor hygiene among them [17]. Detailed medical records on their health status are lacking, as they do not have much faith in modern therapy but resort to traditional therapy and self-healing management. The local authority consequently is facing challenges in strategizing health care plans for these communities.

We therefore undertake this study to develop a disease-risk predictive model in an attempt to characterize the individuals with cardiovascular disease versus healthy individuals based on the metabolite profiles. Orang Asli are chosen as subjects in this study due to several reasons. Based on the findings of several qualitative studies conducted in the 1970’s and 1990’s, low or unreported cases of coronary artery diseases such as fibrous plaques and complicated lesions were found among the Orang Asli [18-20]. In 2010 and 2012, the Orang Asliwere reported to suffer from diabetes, obesity and metabolic syndromes [21,22] but no report on cardiovascular diseases was reported thus far. From the communication with the local Orang Asli, there were many cases of sudden death with unknown reasons which we believed could be cardiovascular-related events, such as myocardial infarction.

In addition, as blood sampling from the Orang Asli is difficult and challenging and routine biochemical analyses require higher volume of blood sample for determination of kidney function, liver function and cardiovascular status of the subject for diagnosis of diseases, metabolomics is believed to be more appropriate. We had previously reported the use of global metabolomics methods that allow us to determine the metabotypes of the patients with 200 microliters of serum [23].

## Materials and methods

### Subjects

The project was approved by the local constitutional Human Research Ethics Committee of UniversitiTeknologi MARA and Department of Orang Asli Development (JAKOA). Good clinical practice and Declaration of Helsinki were followed closely in the study. Prior to sample collection, the purpose and protocol of the study were explained to the subjects and written informed consents were obtained. All the subjects recruited were of 18-65 years old. Clinical interviews were done by medical officers. Demographic data were also collected. The subjects were classified into three groups: urban population of cardiovascular patients with history of myocardial infarction (MI; n = 31) and healthy volunteers (HT; n = 23), and self-claimed healthy Orang Asli (OA; n = 34). The thirty-one (31) patients were recruited from the local hospital. Any patient with other defined illness and chronic disease was excluded. Thirty-four (34) Orang Asli recruited do not have any known diseases. All the healthy volunteers have no current or previous history of cardiovascular disease or any chronic disease. Routine biochemistry test including liver function test, kidney function test were performed to ensure they have no abnormality in these tests. They were not taking any supplement or medication one week before blood withdrawal.

### Sample preparation

Serum samples were used in our study. Protein precipitation and purification of the samples were performed by adding 600 μl of cold acetonitrile into 200 μl of serum. The samples were then centrifuged at 10000*xg* for 10 minutes at 4°C. The supernatant was then transferred into a microcentrifuge tube and dried using a vacuum concentrator (Eppendorf, Hamburg, Germany).

### Global metabolomics using LC/MS-QTOF analysis

Freshly prepared samples were reconstituted with mobile phase (A 95% dH_2_O: B 5% ACN). Two microliters of the samples were injected and analysed by LC/MS-QTOF (model 6520 Agilent Technologies, SA, USA) using a ZORBAX Eclipse Plus C18 column (100 mm × 2.1 mm × 1.8 μm, Agilent Technologies, SA, USA) maintained at 40°C. The system was operated with a flow rate of 0.25 mL/min with solvent A (water with 0.1% formic acid) and solvent B (acetonitrile with 0.1% formic acid). A linear gradient was developed over 36 minutes from 5% to 95% of mobile phase (B). The total run time was 48 minutes for each analysis. Electrospray ionization (ESI) source settings were as follows: V Cap 4000 V, skimmer 65 V and fragmentor 125 V. The nebulizer was set at 45 psig and the nitrogen drying gas was set at a flow rate of 12 L/min. The drying gas temperature was maintained at 350°C. Data were collected in a positive ESI mode and in full scan mode from 100 to 1000 *m/z*. During the analysis, two reference masses of 121.0509 *m/z* (C_5_H_4_N_4_) and 922.0098 *m/z* (C_18_H_18_O_6_N_3_P_3_F_24_) were continuously injected to allow constant accurate mass correction.

Each sample was analyzed in four replicates. Quality control (QC) samples were prepared by pooling aliquots of all the samples analysed. Each QC sample was analysed independently. QC samples were injected at the beginning, middle and end of the run to check the system’s stability and performance. The performance of QC was evaluated by calculating the distribution of relative standard deviation (RSD) of metabolites that are constantly present in 80% of the pooled samples (see Additional file 1).

### Identification of metabolites

Potential metabolite markers for the patients were confirmed by targeted MS/MS approach (model 6520 Agilent Technologies, SA,USA). The experiments were repeated with chromatographic conditions identical to the primary global metabolomics analysis. Ions were targeted using collision-induced dissociation fragmentation (20 V) based on the previously determined accurate mass. Accurate mass data and isotopic distributions for the precursor and product ions were studied and compared to the spectral data of reference compounds from MassBank database (www.massbank.jp). The spectra for the targeted compounds and its product ion were viewed in Additional file 2.

### Data mining and statistical analysis

**Figure 1:**
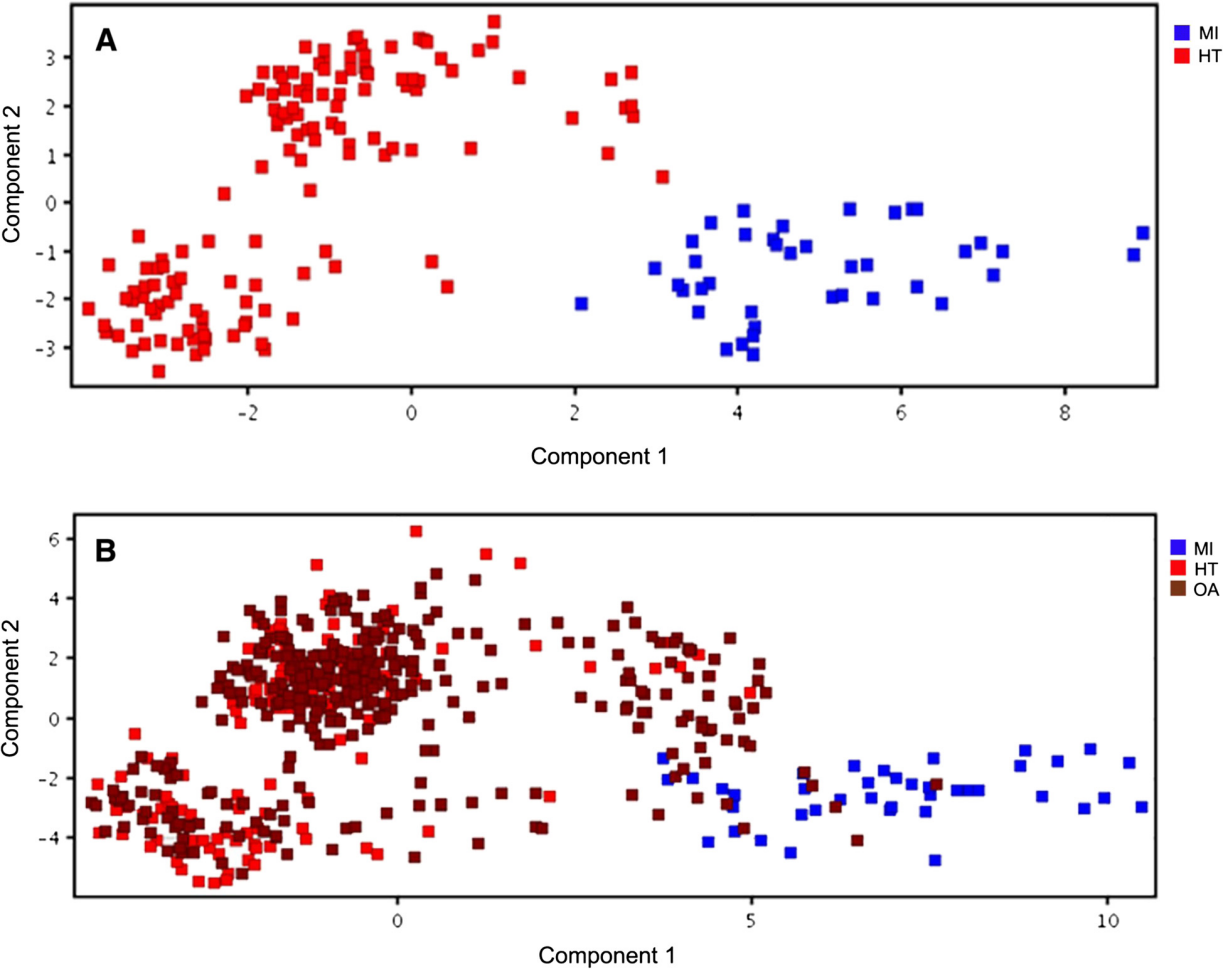
**Principal component analysis of MI and HT (A) and MI, OA and HT (B).** PCA represents a separation of MI and HT group **(A)**. **B** shows that several Orang Asli were clustered within the MI.

The data processing and data analysis were as described and modified from previous study [24,25]. Metabolite extraction and data mining were performed using Agilent MassHunterQualitative Analysis software. Background noise were excluded and removed from the analysis. For statistical analysis, the datasets were imported into Agilent software package (Mass Profiler Professional) for univariate and multivariate analysis. All data were normalized. Differences of metabolites in patients and healthy volunteers were evaluated using *t*-test (*p*< 0.005). Principal component analysis (PCA) was performed to investigate and visualize the profiles of serum metabolites between patients and healthy volunteers (Figure 1). Fold change analysis and volcano plot analysis were performed to determine the metabolites that passed two-fold changes with *p*< 0.005. Area Under Receiver Operating Characteristic (AUROC) curve analysis of metabolites which were significantly different were done to validate the results. The biomarkers were selected if the value of area under is more than 0.7. Pathway analysis of the metabolites was performed with Metabolomics Pathway Analysis (MetPA; http://metpa.metabolomics.ca).

### Prediction of metabotypes

**Figure 2:**
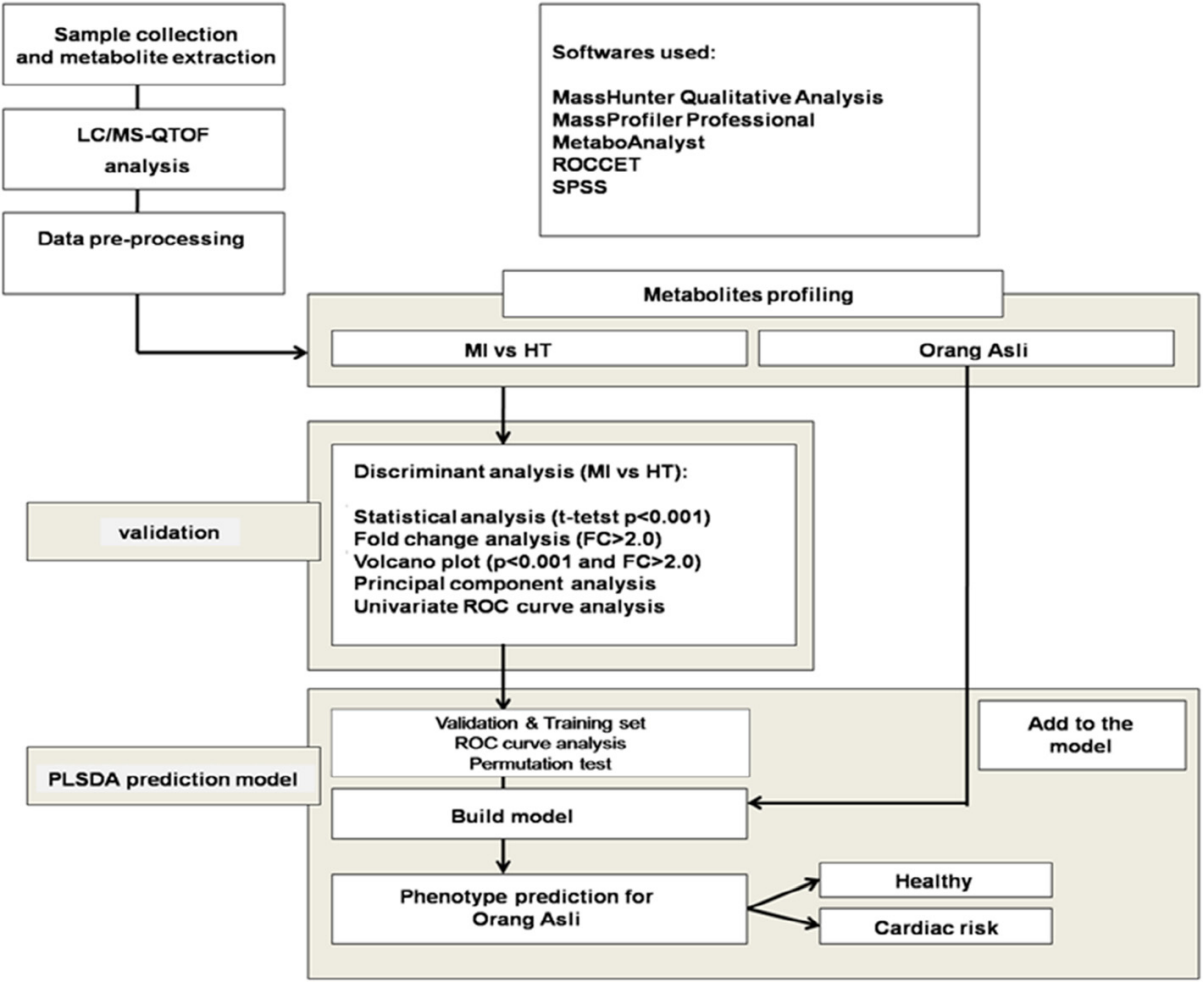
**Workflow for prediction of phenotype among the OA from the serum metabolite profiles.** The workflow was divided into three parts; validation, training and prediction. The OA data were added to the training model [data of healthy volunteers (HT) and patients (MI)] in order to predict their phenotype according to the metabolite profiles and identified biomarkers.

Prediction of metabotypes (healthy or cardiovascular disease traits) among the Orang Asli was done using the multivariate supervised Partial Least Square-Discriminant Analysis (PLSDA). Workflow of the prediction model is as shown in Figure 2. The model was constructed using the biomarkers differentially expressed between the patients and healthy volunteers. Cross-validation and permutation tests (p < 0.001) were performed to validate the accuracy of the constructed model with at least 100 of permutations for each test. For the cross validation, 1/3 of the samples of each group were used in the training analysis, while another 2/3 were used for the validation analysis. Metabotype of the Orang Asli were predicted from the model based on their metabolite profiles. To verify the validity and strength of the model, Receiver Operating Characteristic (ROC) curve analysis was performed to quantify the diagnostic performance of the fourteen candidate metabolites. Multivariate analysis of PLSDA, Support Vector Machine (SVM) and Random Forest (RF) were performed to support the result.

## Results

### Metabolite profiles and biomarkers discovery

A total of eighty-six (86) metabolites were annotated and identified from an average of 1658 spectra detected from LC/MS-QTOF analysis. The identities of the metabolites were determined using the metabolite database (METLIN, Human Metabolome Database, LipidMaps and KEGG). Significant analysis (*t*-test, *p*< 0.005) and fold change analysis showed that twenty-four (24) metabolites were significantly different between patients and healthy volunteers, with at least two-fold differences (Table 1).

**Table 1:** **Lists of metabolites differentially expressed among the patients (MI) and healthy volunteers (HT)**

**Class of metabolite**	**Metabolite**	***p*** **-value (** ***p*** **= 0.005)**	**Log fold change**	**AUC**	**Sensitivity**	**Specificity**
Lipids	8,11,14-nonadecatriynoic acid	3.72E-11	-13.01	0.727	0.8	0.8
	18-oxo-nonadecanoic acid	1.17E-05	-3.30	0.691	0.8	0.6
	5,8-heptadecadiynoic acid	1.28E-18	-4.29	0.754	0.9	0.7
	19-methyl-heneicosanoic acid	4.37E-08	-2.78	0.680	0.7	0.5
	Methyl 12,13-epoxy-9,15-octadecadienoic acid	4.61E-05	1.14	0.776	0.7	0.7
	5,8,11,14-Docosatetraynoic acid	7.49E-10	1.44	0.861	0.8	0.8
	2E,5Z,8Z,11Z,14Z-eicosapentaenoic acid	4.66E-06	5.20	0.909	0.8	0.8
	14-methyl-8-hexadecen-1-ol	1.07E-07	5.52	0.689	0.5	0.8
	15(S)-HETE	6.18E-15	11.04	0.997	1.0	1.0
	Prostaglandin E2	2.58E-06	2.86	0.910	0.8	0.9
	Vitamin K1 2,3-epoxide	1.11E-05	1.74	0.679	0.6	0.8
	24,25-Dihydroxyvitamin D	3.48E-06	-2.07	0.874	0.8	0.8
	21-Deoxycortisol	3.97E-09	-2.90	0.688	0.7	0.7
	C22 Sulfatide	7.06E-11	-8.73	0.766	0.8	0.8
	1-(9Z-heptadecenoyl)-2-docosanoyl-sn-glycerol	2.33E-03	-1.95	0.655	0.8	0.5
	1-(9Z,12Z-heptadecadienoyl)-2-(9Z,12Z-octadecadienoyl)-sn-glycerol	1.72E-04	2.63	0.681	0.8	0.7
	Phosphorylcholine	5.31E-17	14.04	0.995	1.0	1.0
	GPCho(16:1(9E)/0:0)	1.61E-05	5.22	0.737	0.9	0.6
	GPCho(16:1(9Z)/2:0)	3.39E-05	1.96	0.881	0.8	0.8
	GPCho(O-18:1(9Z)/0:0)	2.10E-04	2.23	0.869	0.8	0.8
Others	Indoleacetaldehyde	4.05E-07	-5.96	0.751	0.9	0.8
	Inosine	1.20E-04	-4.85	0.652	0.6	0.9
	Biliverdin IX	1.83E-06	-3.51	0.665	0.8	0.6
	L-Urobilinogen	1.48E-05	5.32	0.635	0.8	0.8

Significant analysis was performed using t-test unequal variance (*p*<0.005). The (-) Fold change value indicates the down regulation of the metabolite in MI, while (+) fold change value indicates the up regulation of metabolite in the MI when compared to HT.

**Figure 3:**
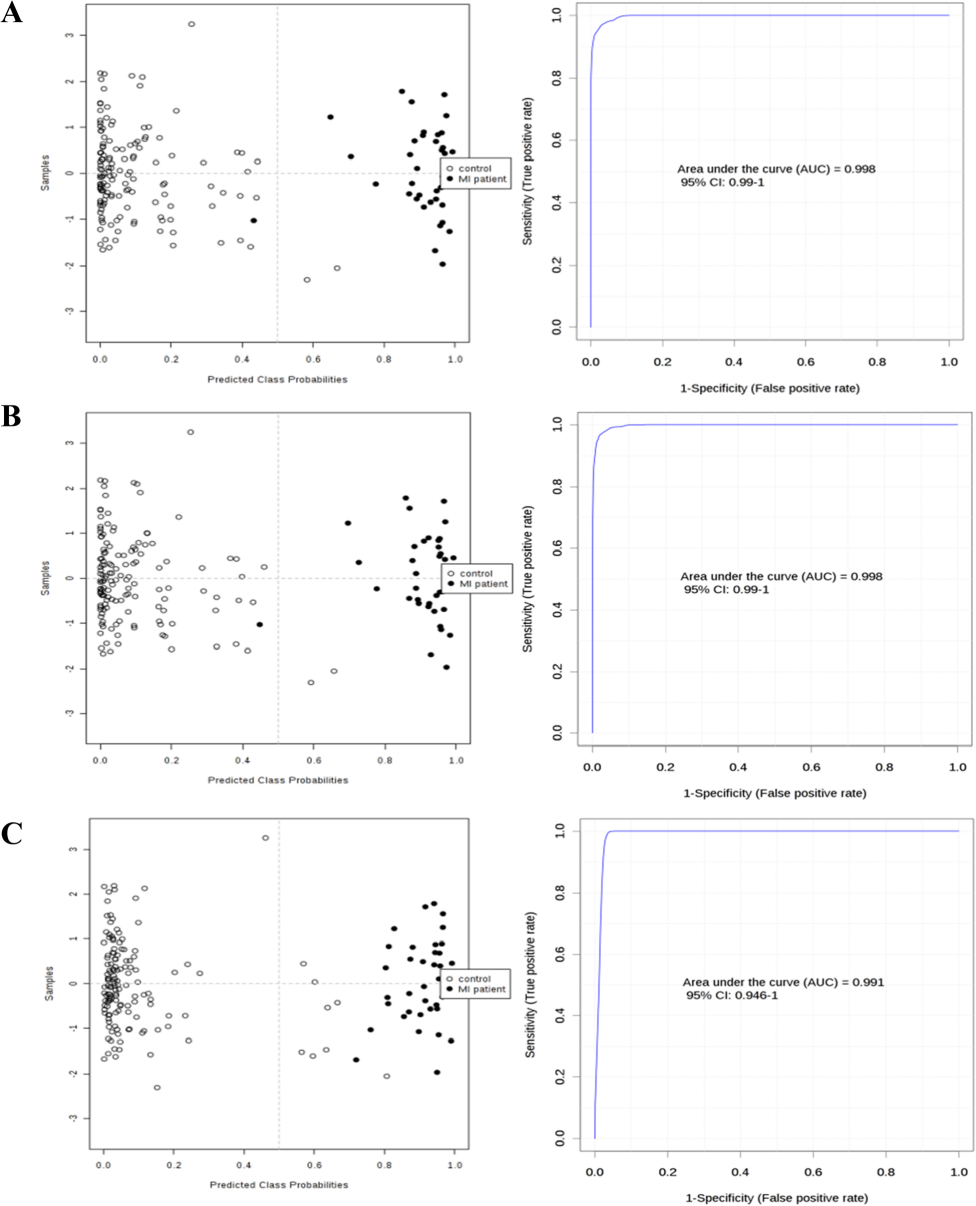
**Quantitative evaluation of the diagnostic performance for putative biomarkers.** Receiver operating characteristic (ROC) curve analysis was performed to quantify the diagnostic performance of the nineteen candidate metabolites using PLSDA **(A)**, Support Vector Machine (SVM) **(B)** and Random Forest (RF) **(C)**. The biomarkers were capable of discriminating the MI from HT with area under curve (AUC) of 0.998, 0.998 and 0.991, with average accuracy of 0.947, 0.961 and 0.963, respectively.

Further analysis of the potential biomarkers was performed using the AUROC curve analysis. As a result, fourteen (14) metabolites were shown to be significantly different between patients and healthy volunteers with AUC of at least 0.7. Two (2) metabolites; 15S-HETE (AUC = 0.997) and phosphorylcholine (AUC = 0.995) with the highest potential of being the serum biomarkers for patients were identified. Both metabolites were significantly up-regulated in the patients as compared to the healthy volunteers. The performances of the metabolites as potential biomarkers were validated using PLSDA, Support Vector Machine (SVM) and Random Forest (RF). Multivariate AUROC analysis of PLSDA, SVM and RF were performed to validate the performance of the biomarkers and the results were supported by multivariate ROC curve analysis (Figure 3). The biomarkers were capable of discriminating patients from the healthy volunteers with the AUC of 0.998, 0.998 and 0.991, and average accuracy of 0.947, 0.961 and 0.963, respectively. These results denoted that the fourteen (14) serum metabolites were potential classifiers between the patients and healthy volunteers.

Two main metabolic pathways were found to be significantly altered among patients when compared to the healthy volunteers. They are metabolisms of lipids (glycerophospholipid metabolism, fatty acid metabolism, sphingolipid metabolism, linoleic acid metabolism, arachidonic acid metabolism and glycerolipid metabolism) and amino acids (see Additional file 3).

### Metabotypes prediction of the Orang Asli

**Figure 4:**
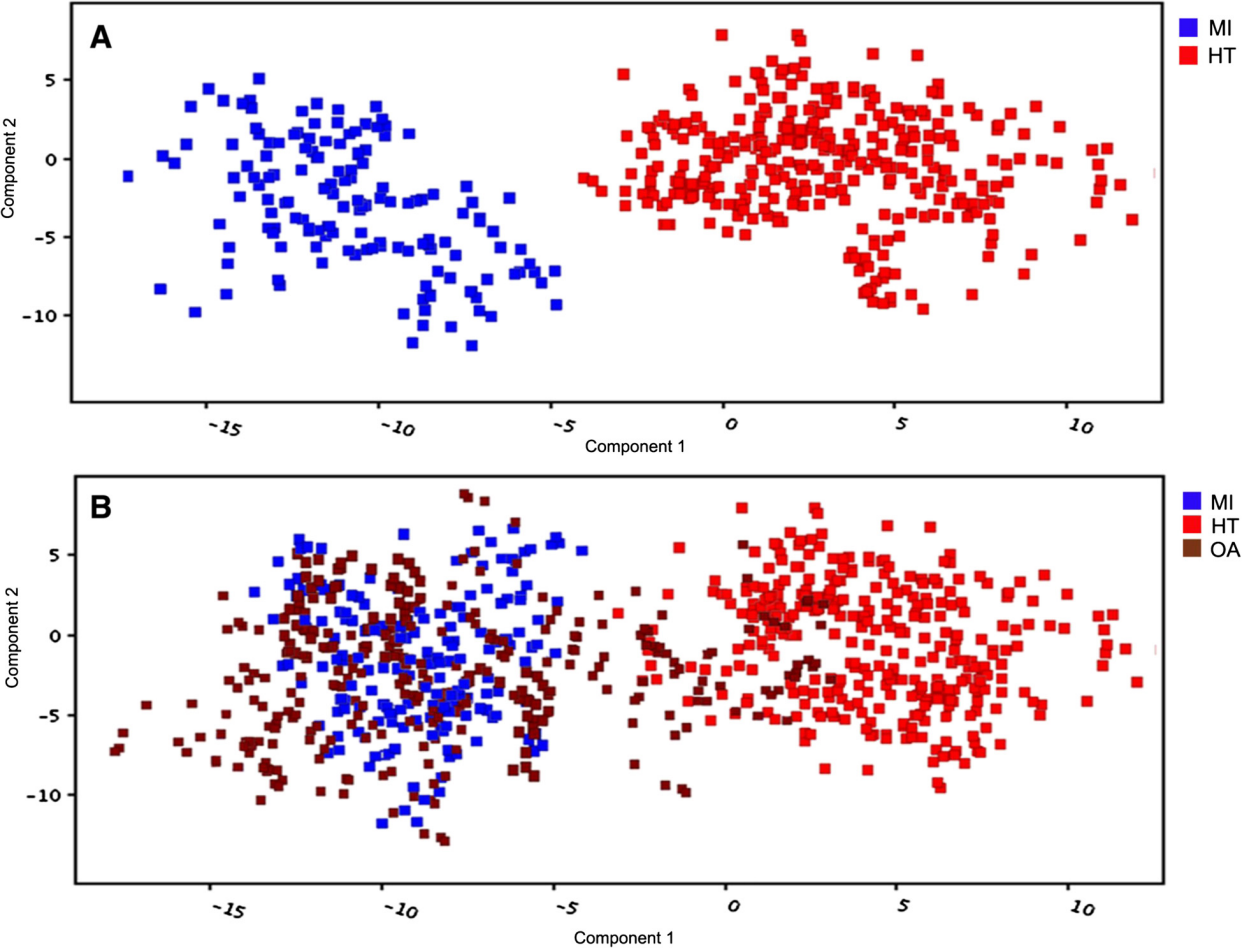
**Score plot of the PLSDA prediction model of the MI, HT and OA using identified biomarkers. A** shows the PLSDA of MI and HT while **B** shows the PLSDA of MI, HT and OA. OA data were added to the model for prediction resulted that several OA subjects were predicted to have similar profiles with patients with cardiovascular diseases. The model was constructed according to the metabolites which have high specificity and sensitivity (AUC >0.7).

A cross-validated PLSDA model with satisfactory discriminating ability was constructed using the fourteen (14) metabolites to assess the metabolic differences between the patients and healthy volunteers (Figure 4). Validation was obtained from permutation test (n = 100) with *p*< 0.05. The prediction model demonstrated that twenty-seven (27) Orang Asli were predicted to have healthy status, while the other seven subjects were predicted to have profiles similar to the patients. Validation using ROC curve analysis showed an AUC value of 0.998 as the confidence value of the model.

## Discussions

PLSDA is widely used as a statistical tool for clustering. A wide range of metabolomics data can be used to predict and classify unknown samples using PLSDA approach. The predictive and orthogonal components supported by PLSDA facilitate the interpretation of class discrimination and the prediction of class membership of unknown samples. Class prediction using metabolomics data can be used for early diagnosis, prognosis and treatment of diseases. By using the PLSDA prediction model, we were able to predict the metabotypes of the Orang Asli according to their metabolite profiles with an average accuracy of 0.998. The prediction model had classified seven (7) Orang Asli with the cardiovascular patients. Majority of the Orang Asli were predicted to be healthy. This is in accordance with what the individuals had claimed and similar with the results obtained from biochemical analysis. The biochemistry profiles of the seven (7) subjects showed that their lipid profiles were in the abnormal ranges which were associated with cardiovascular risk. They include low levels of HDL (<1.3 mmol/L), very high level of LDL, high level of triglycerides (>1.3 mmol/L) and high level of cholesterol (>5.2 mmol/L) (see Additional file 4). However, the advantage of using metabolomics platform is that only a small volume of blood is required as compared to the routine biochemical assays which require larger volume of blood samples. This shows that LC/MS-QTOF is a sensitive method and appropriate in studies alike, where blood samples are difficult to obtain. This is especially important in conserved communities like the Orang Asli, where many of them refused to provide large volume of blood for medical examination.

Lipid profiles including triglycerides, total cholesterol and HDL and LDL are clinically used as the parameters for detection of several diseases. Lipid abnormalities are associated with the incidence of coronary heart disease and other cardiovascular diseases [26]. High LDL level in the body can increase the risk of cardiac disease, whilst low HDL level is an independent cardiovascular risk factor. The increase of HDL by only 1 mg/dL can lead to risk reduction of the disease. The contents of HDL are proteins and lipids, including various glycerophospholipids, cholesteryl esters, sphingomyelins, free fatty acids, monoacylglycerols, diacylglycerols, triacylglycerols, and different sphingolipids [27]. These compounds can be profiled using global and targeted metabolomics approach. HDL has the protective antiatherogenic properties, which are independent of their involvement in cholesterol metabolism and it also reduces oxidation and vascular inflammation [28]. The ability to profile lipid metabolism in clinical setting is thought to be useful as dysfunction of lipid metabolism is associated with inflammation and oxidative stress which significantly increased the risk of atherosclerotic cardiovascular diseases [29].

Of the fourteen potential biomarkers, we had detected two metabolites with the highest value of AUC, sensitivity and specificity. They are 15(S)-HETE and phosphorylcholine. 15(S)-HETE is a metabolite involved in the metabolism of arachidonic acid and was found to be significantly elevated in the patients as compared to the healthy volunteers. Our findings were supported by the earlier findings which showed that the elevated HETE was found in the patients with heart diseases [30,31]. In addition, pathway enrichment analysis also showed that arachidonic acid metabolism is one of the pathways that are involved in the alteration of the patient's metabolic pathway. This metabolite is derived from omega-3 or omega-6 polyunsaturated fatty acids. At the same time, it is involved in the production of other metabolites, including prostaglandins, thromboxanes, leukotrienes, epoxyeicosatrienoic acid and oxo-fatty acids. In cardiovascular diseases, eicosanoids and fatty acids are probably involved in the oxidative stress, inflammation, plaque formation and pathogenesis [32,33], thus further strengthening the relevance of 15(S)-HETE as a marker.

Another potential biomarker, phosphorylcholine, was also found to be significantly elevated in the patients. This metabolite was involved in two metabolic pathways, which are the glycine, serine and threonine metabolism and glycerophospholipid metabolism pathway. In cardiovascular event, phosphorylcholine is a common epitope that can be found in oxidized LDL. It plays an important role in the post infarction setting by activating the endothelial cells to recruit monocytes into the vascular wall leading to inflammation and atherogenesis [34]. Phosphorylcholine epitope is recognized by many parts of the innate immune system, including B-lymphocytes, which release natural antibodies such as IgM that work against phosphorylcholine [35]. Therefore the increased level of phosphorylcholine denotes the process of inflammation and atherogenesis among the patients and may be a useful marker to monitor patients.

Our study also observed a significant decreased of 24,25-Dihydroxyvitamin D derivatives in patients as compared to healthy volunteers. This result was supported by an earlier study, which also observed a low level of vitamin D in the heart disease patients [36]. It has been reported that vitamin D deficiency was associated with high BMI, cardiovascular diseases and obesity [37]. As most of the Orang Asli are poorer in nutritional status and have adopted sedentary lifestyles due to the relocation programme, therefore their risks to CVD are increased.

Lower abundance of purine metabolites, specifically inosine were observed among the patients in this study. Inosine is the precursor metabolite of deoxyadenosine and uric acid. It is formed from the binding of hypoxanthine and ribose. It is the intermediate product during the degradation of purines and purine nucleosides to uric acid. This is interestingly in accordance with the previous study in an ischemic mouse heart model which showed that the endogenous inosine might be a potential biomarker of initial cardiac ischaemia before cardiac tissue necrosis [38]. It is believed that adenosine and inosine have the cardioprotective effects. It was also reported that increase production of adenosine and inosine by ischemic myocardium supports the hypothesis that these cardiac nucleosides may have important roles in the adaptation of coronary blood flow in human coronary artery disease [39].

We also observed a significant difference of sphingolipids metabolism between the patients and healthy volunteers. Sphingolipids are the minor components of lipid in most mammalian cells. It is an important bioactive lipid species which is involved in the pathogenesis of cardiovascular diseases [40]. A metabolite of sphingolipids; C22 sulfatide was found to be significantly lower in the patients as compared to the healthy volunteers. This result was supported by Hu *et al*. [41] which reported the ability of low level of serum sulfatides in initiating the development of CVD. However, there are no significant correlation of low sulfatide level with the measurements of HDL, LDL, cholesterol and triacylglycerol.

## Conclusion

Metabolomics-PLSDA risk prediction model was validated and used to classify individuals based on their metabotypes. In this study, the model was used for prediction of CVD risks among the Orang Asli. Some of the Orang Asli with self-claimed healthy status were shown to have metabolite profiles similar to the patients. This shows that metabolomics is a useful alternative to the routine biochemistry tests. The metabolites differentiating the individuals into clustered groups were shown to be good biomarkers with high AUROC values, however, further validations are required before their use in clinical practise. More patients related metabolomics database is needed to realise the use of metabolomics which may be more cost effective in providing the wholesome metabolism picture of a patient at real time. Concerted efforts from researchers are needed in the setting up of clinical useful metabolome database.

## Additional files

Additional file 1:
**Relative standard deviation of metabolites of pooled samples.**
Additional file 2:
**MS/MS result for several targeted metabolites.**
Additional file 3:
**Result from pathway analysis using MetPA.**
Additional file 4:
**Baseline characteristics for the patients (MI), healthy volunteers (HT) and Orang Asli (OA).**

## Abbreviations

MI: Myocardial infarction
HT: Healthy volunteers
OA: Orang Asli
CVD: Cardiovascular disease
PLSDA: Partial least square discriminant analysis
PCA: Principal component analysis
