# Supplementary material for: Metabolomics and partial least square discriminant analysis to predict history of myocardial infarction of self-claimed healthy subjects: validity and feasibility for clinical practice
Source: J Clin Bioinforma. 2015 Mar 13;5:3. doi: 10.1186/s13336-015-0018-4 (PMC4371619; doi:10.1186/s13336-015-0018-4)
Supplement: Additional file 1: — Relative standard deviation of metabolites of pooled samples. [file 13336_2015_18_MOESM1_ESM.docx]

**Additional file 1.**

Relative standard deviation of metabolites of pooled samples

| Compound | Mass Diff (ppm) | | | RT diff (min) | | |
| --- | --- | --- | --- | --- | --- | --- |
|  | Mean | St. Dev | RSD (%) | Mean | St. Dev | RSD (%) |
| Propionyl-L-carnitine | 0.4632 | 0.0188 | 4.06 | 0.0245 | 0.0026 | 8.00 |
| cis-5-Tetradecenoylcarnitine | 0.7082 | 0.1024 | 14.46 | 0.0580 | 0.0114 | 18.99 |
| GPEtn(18:0 /0:0) | 1.4586 | 0.1983 | 13.60 | 1.4574 | 0.0732 | 5.01 |
| 10-hydroxy-2E,8Z-Decadiene-4,6-diynoic acid | 0.5539 | 0.0109 | 1.97 | 0.1198 | 0.2077 | 17.27 |
| D-Glucose | 0.0628 | 0.0006 | 0.96 | 1.0386 | 0.0219 | 2.01 |
| palmitoylcarnitine | 0.0430 | 0.0008 | 1.86 | 0.7760 | 0.0372 | 4.79 |
| Oleamide | 0.2714 | 0.0008 | 0.29 | 0.0568 | 0.0030 | 5.28 |
| Leucine | 0.0005 | 6.74E-05 | 13.48 | 0.0259 | 0.0006 | 2.32 |
| L-Octanoylcarnitine | 0.0081 | 0.0004 | 4.94 | 0.0434 | 0.0083 | 19.12 |
| 1-Aminocyclohexanecarboxylic acid | 0.0043 | 0.0006 | 13.95 | 0.0039 | 0.0002 | 5.13 |
| Uric acid | 0.0080 | 0.0007 | 8.75 | 0.0306 | 0.0162 | 0.65 |
| indoleacrylic acid | 0.0039 | 0.0004 | 10.26 | 0.0594 | 0.0039 | 6.56 |
| Dihydrosphingosine | 0.0081 | 0.0015 | 18.52 | 0.2151 | 0.0333 | 15.48 |
| C16 Sphinganine | 0.0076 | 0.0008 | 10.53 | 0.4268 | 0.0567 | 13.29 |
| C17 Sphinganine | 0.0254 | 0.0007 | 2.76 | 0.818 | 0.0475 | 5.8 |
