# Supplementary material for: Metabolomics and partial least square discriminant analysis to predict history of myocardial infarction of self-claimed healthy subjects: validity and feasibility for clinical practice
Source: J Clin Bioinforma. 2015 Mar 13;5:3. doi: 10.1186/s13336-015-0018-4 (PMC4371619; doi:10.1186/s13336-015-0018-4)
Supplement: Additional file 2: — MS/MS result for several targeted metabolites. [file 13336_2015_18_MOESM2_ESM.doc]

| **A** | **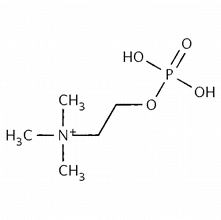** |
| --- | --- |
| **B** | **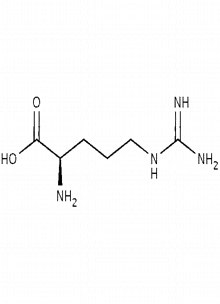** |
| **C** | **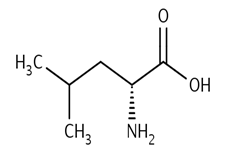** |

**Additional file 2.** MS/MS result for several targeted metabolites. CID spectra, fragmentation patterns, product ions and chemical structures of the [M + H]+ ions of phosphorylcholine (A), arginine (B) and leucine (C).
