# Supplementary material for: Metabolomics and partial least square discriminant analysis to predict history of myocardial infarction of self-claimed healthy subjects: validity and feasibility for clinical practice
Source: J Clin Bioinforma. 2015 Mar 13;5:3. doi: 10.1186/s13336-015-0018-4 (PMC4371619; doi:10.1186/s13336-015-0018-4)
Supplement: Additional file 3: — Result from pathway analysis using MetPA. [file 13336_2015_18_MOESM3_ESM.doc]

**Additional file 3.**

Result from pathway analysis using MetPA.

| Pathway name | Total | Hits | p-value | Impact |
| --- | --- | --- | --- | --- |
| Glycerophospholipid metabolism | 39 | 3 | 4.20E-37 | 0.06 |
| Fatty acid biosynthesis | 49 | 1 | 8.61E-16 | 0.00 |
| Amino sugar and nucleotide sugar metabolism | 88 | 1 | 8.33E-12 | 0.00 |
| Sphingolipid metabolism | 25 | 2 | 2.04E-11 | 0.14 |
| Tryptophan metabolism | 79 | 1 | 1.06E-06 | 0.01 |
| Linoleic acid metabolism | 15 | 3 | 2.10E-05 | 0.66 |
| Arachidonic acid metabolism | 62 | 2 | 1.53E-04 | 0.03 |
| Purine metabolism | 92 | 2 | 3.61E-04 | 0.01 |
| Glycerolipid metabolism | 32 | 3 | 3.58E-03 | 0.12 |

*****The Hits is number of matched metabolites from the uploaded data to the database.
