# Supplementary material for: Metabolomics and partial least square discriminant analysis to predict history of myocardial infarction of self-claimed healthy subjects: validity and feasibility for clinical practice
Source: J Clin Bioinforma. 2015 Mar 13;5:3. doi: 10.1186/s13336-015-0018-4 (PMC4371619; doi:10.1186/s13336-015-0018-4)
Supplement: Additional file 4: — Baseline characteristics for the patients (MI), healthy volunteers (HT) and Orang Asli (OA). [file 13336_2015_18_MOESM4_ESM.doc]

**Additional file 4.**

Demographic and clinical data of patients (MI), healthy volunteers (HT) and Orang Asli (OA).

| Characteristic | Normal range | MI | OA | HT | p-value  (p<0.05) |
| --- | --- | --- | --- | --- | --- |
| Number of subjects (n) |  | 31 | 34 | 23 |  |
| Age (mean, ±SD) |  | 57.47 (±1.271) | 34.28 (±1.67) | 26.00 (±1.561) | <0.00* |
| BMI  (kg/m2) | 18.5 - 23.9 | 24.73  (18.82-37.29) | 21.06  (16.30-36.30) | 21.55  (16.50-36.30) | 0.000** |
| Medication |  | 31/31 | - | - |  |
| Systolic pressure  (mmHg) |  | 128  (103-176) | 126  (99-164) | 122.5  (102-154) | 0.241** |
| Diastolic pressure  (mmHg) |  | 77  (40-101) | 79  (61-106) | 73.50  (58-103) | 0.159** |
| Pulse rate |  | 72.94 (±2.57) | 86.69 (±2.14) | 76.39 (±2.13) | 0.127* |
| Cholesterol  (mmol/L) | <5.2 | 4.70  (3.00-6.78) | 4.40  (1.70-6.90) | 4.20  (3.20-6.74) | 0.002** |
| HDL  (mmol/L) | >1.3 | 1.15  (0.76-1.76) | 1.00  (0.40-2.00) | 1.70  (1.20-2.20) | <0.000** |
| LDL  (mmol/L) | <2.6 | 3.00  (1.71-5.06) | 2.60  (0.60-4.90) | 3.40  (1.70-5.70) | 0.024** |
| TG  (mmol/L) | <1.7 | 1.24  (0.50-3.29) | 1.20  (0.40-4.30) | 0.70  (0.40-1.80) | 0.002** |
| Urea  (mmol/L) | <8.3 | 6.30  (2.70-10.50) | 3.20  (1.40-6.30) | 3.90  (2.40-5.30) | <0.000** |
| Creatinine  (umol/L) | 44.0-106.0 | 97.00  (60.00-170.00) | 75.50  (52.00-105.00) | 77.00  (47.00-104.00) | <0.000** |
| Sodium  (mmol/L) | 136-145 | 139.00  (136.0-142.0) | 139.75  (134.0-144.0) | 139.00  (136.0-144.0) | 0.299** |
| Potassium  (mmol/L) | 3.5-5.1 | 4.20  (3.40-4.90) | 4.10  (3.10-5.30) | 4.30  (3.60-5.40) | 0.043** |
| Albumin  (g/L) | 35.0-52.0 | 44.00  (29.00-47.00) | 45.50  (41.20-54.70) | 51.00  (46.60-58.40) | <0.000** |
| ALP  (U/L) | <33.0 | 59.00  (15.00-159.00) | 79.70  (55.30-210.10) | 66.70  (54.10-140.00) | 0.002** |
| ALT  (U/L) | 35.0-130.0 | 29.00  (10.00-78.00) | 16.25  (8.50-96.80) | 16.30  (5.60-47.80) | 0.003** |
| Total bilirubin  (umol/L) | <21.0 | 12.00  (7.00-25.00) | 5.40  (1.80-20.00) | 10.60  (4.10-23.40) | <0.000** |
| Total protein  (g/L) | 66.0-87.0 | 72.00  (61.00-86.00) | 75.30  (68.00-90.30) | 76.40  (69.70-91.60) | 0.016** |

*statistical analysis was performed using t-test with p<0.05

**statistical analysis was performed using non-parametric test Kruskal-Wallis H with p<0.05.
